# Factors associated with poor glycemic control among adult patients with type 2 diabetes mellitus in Gamo and Gofa zone public hospitals, Southern Ethiopia: A case-control study

**DOI:** 10.1371/journal.pone.0276678

**Published:** 2023-03-10

**Authors:** Firehiwot Dawite, Meseret Girma, Tamiru Shibiru, Etenesh Kefelew, Tadiwos Hailu, Rodas Temesgen, Getachew Abebe

**Affiliations:** 1 School of Public Health, College of Medicine and Health Science, Arba Minch University, Arba Minch, Ethiopia; 2 School of Medicine, College of Medicine and Health Science, Arba Minch University, Arba Minch, Ethiopia; 3 Department of Anatomy, College of Medicine and Health Science, Arba Minch University, Arba Minch, Ethiopia; Federal Medical Centre Umuahia, NIGERIA

## Abstract

**Background:**

Diabetes mellitus is a serious global public health problem that affects the whole life of people in terms of their biological, psychological, and social effects. Complications and death from diabetes occur from poorly controlled blood glucose levels. Thus, dealing with glycemic control is essential for controlling the development of devastating acute and chronic complications related to diabetes. Therefore, this study aims to assess factors associated with poor glycemic control among type2 diabetes patients in public hospitals of Gamo and Gofa zone southern, Ethiopia, 2021.

**Methods:**

An institution-based unmatched case-control study was employed among 312 randomly selected participants using a pre-tested, interviewer-administered, and structured questionnaire. Bivariate and multivariable logistic regression analysis was conducted to identify factors associated with poor glycemic control using IBM SPSS version 25. The strength of association was assessed by using an Adjusted odds ratio (AOR) with a 95% confidence interval (CI).

**Result:**

Factors associated with poor glycemic control based on multivariable analysis were, having comorbidity (AOR = 2.35, 95% CI (1.39–3.95)), adhering to dietary recommendations (AOR = 0.31, 95% CI (089–0.51)), poor social support (AOR = 3.31, 95% CI (1.59–6.85)), physical exercise (AOR = 1.86 95% CI (1.11–3.12)), and having poly-pharmacy (AOR = 2.83, 95% CI (1.39–5.74)).

**Conclusion and recommendation:**

This study indicated a significant association of comorbidity, physical exercise, poly-pharmacy, low social support, and adherence to dietary recommendations with poor glycemic control. We suggest that the health care providers and concerned bodies encourage patients to have regular check-ups and work on providing necessary social support.

## Introduction

Non-communicable diseases (NCDs), such as cardiovascular diseases, cancers, diabetes, and chronic respiratory diseases, are now the leading cause of death in most regions of the world [1]. Diabetes is a serious, chronic disease that occurs either when the pancreas does not produce enough insulin (a hormone that regulates blood glucose), or when the body cannot effectively use the insulin it produces [2]. Type 2 diabetes mellitus (T2DM), previously referred to as “noninsulin-dependent diabetes” or “adult-onset diabetes,” accounts for 90–95% of all diabetes. This form encompasses individuals who have relative insulin deficiency and peripheral insulin resistance [3]. The global diabetes prevalence in 20–79-year-olds in 2021 was estimated to be 10.5% (536.6 million people), rising to 12.2% (783.2 million) in 2045 [4]. The burden of diabetes mellitus (DM) is higher in low and middle-income countries like Ethiopia where, the total unmet need for diabetes care is around 77.0%, and rural facilities, particularly in SSA lack access to proper monitoring of glucose like hemoglobin A1c and other key biologic tests [5, 6]. About half (46.2%) of the deaths attributable to diabetes occur in people under the age of 60 years [7]. The Africa Region has the highest (73.1%) proportion of deaths attributable to diabetes in people under the age of 60 years [7]. Furthermore, diabetes carries a significant “double burden” of infectious and chronic diseases in Africa [8]. Diabetes is not fatal if managed effectively, but untreated hyperglycemia results in various multi-organ complications that cause acute and chronic morbidity and death [9]. Poor glycemic control is associated with reduced life expectancy, significant morbidity due to specific diabetes-related microvascular complications, increased risk of macrovascular complications, and diminished quality of life [10].

Despite the availability of a wide range of effective glucose-lowering therapies, approximately half of patients with T2DM in the world do not achieve glycemic targets [11, 12]. A multicenter study conducted in Eastern Europe, Asia, and Latin America showed that 96.4% of study participants had poor glycemic control [13]. similarly, Ethiopia had a consistent and high prevalence of poor glycemic control among diabetic patients ranging from 62.5 in Tigray to 65.5 in the Oromo region [14].

In Ethiopia a cross-sectional study has been conducted on factors associated with poor glycemic control Self-monitoring blood glucose, presence of comorbidities, duration of diabetes mellitus, physical activity, total cholesterol of 200 mg/dl or more, waist-to-hip ratio or and types of anti-diabetic medication were identified as factors significantly associated with of poor glycemic control [15–18].

Diabetes management aims to prevent mortality and complications by optimizing the blood glucose level [19]. Clinical trials have shown that tight blood glucose control correlates with a reduction in those complications in a patient with T2DM [20, 21]. Each 1% reduction in the mean Glycated hemoglobin (HbA1c) is associated with a reduction in risk of 21% for deaths related to diabetes, 14% for myocardial infarction, and 37% for microvascular complications [22]. Even if several studies were conducted on factors associated with poor glycemic control in Ethiopia most of them are in the Amhara and Oromia regions which may not represent the southern region of the country very well, so, therefore, there is limited evidence on determinants of poor glycemic control in the southern region of the country. Additionally, the previous studies did not assess the influence of social support and poly-pharmacy on poor glycemic control very well. As well as this study is new in the research area. So then this study aimed to identify factors associated with poor glycemic control among type 2 diabetes mellitus patients in public hospitals of Gamo and Gofa zone, southern, Ethiopia; besides the association of perceived social support with glycemic control was also investigated.

## Materials and methods

### Study design, setting, and period

This study was conducted in selected three Hospitals in Gamo and Gofa Zones, Southern Ethiopia from March 18 to May 18, 2021. These two Zones are found within the South Nations, Nationalities, and Peoples’ Region (SNNPR) of Ethiopia The administrative center of the two zones, Arba Minch (Gamo zone) and Sawla (Gofa zone) are located at 434 km and 455 km respectively far south of Addis Ababa, the capital city of Ethiopia. The total population of the study area is 2,658,345 in the year 2017/18 as estimated from the 2007 Ethiopian census. There are two general and four primary hospitals providing curative, preventive, and rehabilitative services for the population in the two zones. The hospitals have a total of 2115 diabetic patients who are under follow up and from those 1091 T2DM have a follow-up in the three study hospitals.

### Population

Cases were T2DM patients in the follow-up clinic that were classified as having poor glycemic control by using an average of three consecutive Fasting Plasma Glucose (FPG) levels and who had an average FPG >130mg/dl. Controls were T2DM in the follow-up clinic that were classified as having good glycemic control by using three consecutive average FPG levels and who had FPG 80–130 mg/dl.

The source population; is for cases of all patients in the follow-up clinic with poor glycemic control. To control all T2DM in the follow-up clinic with good glycemic control.

The study population; for cases all T2DM with poor glycemic control came to the follow-up clinic during data collection time. To control all T2DM patients with good glycemic control who came to the follow-up clinic during data collection time.

### Sampling

The sample size was calculated by using Epi info7 software stat calc. The following assumptions were considered 95% confidence interval, 80% power, a 50% expected proportion of DM patients with adequate physical exercise from a study in northwest 2017 Ethiopia who had good glycemic control (control), and a 67.1% expected proportion of DM patients with inadequate physical exercise who had poor glycemic control (cases), and a case to control the ratio of 1:1. Based on the above assumptions, the sample size calculated was 312(156 cases and 156 controls). Among the variables considered, physical exercise was selected as an associated factor variable for poor glycemic control since it gave a maximum sample size. Both cases and controls were selected by employing a systematic random sampling technique with a proportional allocation of samples (26, 27, 29, 40, 67).

### Data collection procedures and instruments

Structured interviewer-administered questionnaires were used after reviewing all the relevant literature, and recorded review and physical measurements were done. The questionnaire had four parts: socio-demographic information, clinical and anthropometric measure data related to behavioral factors, and a perceived social support scale.

The Summary of diabetic self-care activity (SDCA) was used to measure behavioral factors such as adherence to diabetes-related exercise and self-monitoring of blood glucose levels. It was used in previous studies in evaluating adherence to diabetes medication and diabetes diet among DM patients [23, 24]. Moreover, 10 and 8 items Modified Morisky Scale (MMS) were used to measure other behavioral factors such as adherence to medication and diet [23, 24].

**Measurement.** A multidimensional scale of perceived social support (MSPSS) was used to measure perceived social support MSPSS. The scale is composed of 12 items in three groups, each of which is composed of four items regarding family (Items 3, 4, 8, and 11), friend (Items 6, 7, 9, and 12), and a special person (Items 1, 2, 5, and 10) [25]. Each item was graded using a 7-point scale. The subscale score is obtained by adding the scores of four items in each scale, and the total scale score is obtained by adding all the subscale scores. The lowest score from the subscales is 4, and the highest score is 28. Based on the mean score it was categorized as good social support if it is above the mean else poor social support [26].

Other DM-related variables that might influence values of glycemic control were taken from medical history records and these are duration with DM, the presence of comorbidity type of medication currently taken, and FPG level.

#### Anthropometric measurements

Bodyweight and height were measured by a stadiometer (Seca Germany) a portable weight scale machine. Body weight was measured to an accuracy of 0.1 kg by using Seca Germany and Subjects were measured barefoot. Height was measured in, standing upright by a stadiometer Seca Germany to the nearest 0.1c.m. Body mass index (BMI) was calculated as the ratio of weight in kilograms (kg) to the square of height in meters (m2).

#### Waist-to-hip ratio

Waist-to-hip ratio (WHR) was measured after the participants stood with arms at the sides, feet positioned close together, and weight evenly distributed across the feet, the waist circumference (WC) was measured to the nearest 1 cm three times at the approximate midpoint between the lower margin of the last palpable rib and the top of the iliac crest, at the end of a normal exhalation. The mean of the three measurements was calculated and taken at the end. Participants were told to relax and take a few deep, natural breaths before the actual measurement was done to minimize the inward pull of the abdominal contents during the waist measurement. The hip circumference (HC) of the patients was measured three times to the nearest centimeter at the largest circumference of the buttocks. Both hip and waist circumferences were measured with stretch-resistant tape that is wrapped snugly around the participants and the tape was kept level and parallel to the floor at the point of measurement. This protocol of measurement is per the world health organization’s (WHO) Stepwise approach to surveillance [27]. Six trained BSc nurses working in different areas of the study sites were recruited as data collectors and two of them were employed as supervisors for consecutive two months.

### Operational definitions

**Table:** 

**Glycemic control**			Patients were categorized based on the American Diabetic Association (ADA) 2019 guideline recommendation into two groups:[15, 28] after taking the average of three consecutive visits of FPG tests.
			**Good glycemic control:** average fasting plasma glucose **of 80–130 mg/dl.**
			**Poor glycemic control:** average fasting plasma glucose **of > 130 mg/dl.**
**Adherence to exercise**			Patients were considered to adhere to exercise if the mean scored ≥ 4 days by using SDCA physical exercise adherence questionnaire [29].
**Adherence to a diabetic diet**			A patient was considered to adhere to a dietary regimen when he/she scored at least 50% of the total 10-item MMS dietary-related question [30, 31].
**Adherence to diabetic medication**			A patient was considered to adhere to a medication when he/she scores at least 80% of the total 8 items of MMS questions [32].
**Co-Morbidity**			Patients with any chronic disease that coexisted with their diabetes were considered to be co-morbid [15].
**Poly-pharmacy**			Polypharmacy was defined as the use of a combination of ≥5 medications daily. Simultaneous poly-pharmacy: was used to estimate the number of drugs a patient is receiving at any given point in time [15, 33].
**BMI**		>18.5 underweight, 18.5–24.9 normal, 25.0–29.9 overweight, and ≥30 obese according to the WHO classification for BMI [34].	
**Central Abdominal obesity**	Waist-to-hip ratio (central obesity) was considered abnormal if the female participant had more than >0.85 cm of waist circumference and the male participant had more than >1 cm [34].		
**Social support**	From a total of multidimensional social support scales, respondents who scored below the mean were taken as having poor social support and those who scored above the mean were taken as having good social support from a total of multi-dimensional social support scales.		

### Data quality management

The structured questionnaire was developed after reviewing different kinds of literature. Then it was translated from English to Amharic and back-translated into English by the same individual to assure its consistency. Three days of training were given to data collectors and supervisors about the aim, procedure, tool, and ethics before data collection. Whereas pretest was done on patients (5%) on 8 cases and 8 controls at Grease primary hospital and necessary changes were done based on the result of the test. The collected data were reviewed and checked daily for completeness and consistency by the supervisor and principal investigator and ongoing supervision was made.

### Data processing and analysis

Data were recorded in the mobile KOBO toolbox and exported to the SPSS version 25 software package for further management and analysis. Descriptive and analytic statistics were done. Descriptive statistics were used to describe the distribution of explanatory variables among case and control. The findings were presented in narration and tables proportions, and mean and standard deviation. Bi-variable and multivariable analyses using a logistic regression model with odds ratio and its corresponding (CI) 95% confidence interval were done. To identify the significant association variables (p≤0.25) were considered the candidate variables for the multivariable binary logistic regression analysis and finally the strength of association was measured by computing the AOR with a 95% CI. The statistical significance was declared at computing (p≤0.05). And all assumptions of binary logistic regression were checked. Multi-collinearity was assessed by using the variance inflation factor and tolerance. The VIF was <10 and the tolerance was >0.1. Multivariate outliers have been checked by cooked distance and it was < 1 since then there was no problem with a multivariate outlier. In addition assumptions of chi-square were assessed none of the cells has an expected frequency <5. Cross-tabulation was used to summarize descriptive statistics. An odds ratio with 95% CI was used for measuring the strength of the association. P-value < 0.05 was considered as statistically significant. The fitness of the model was checked by Hosmer and Lemeshow goodness of fit test and the p-value of the test were 0.321.

### Ethical considerations

Ethical approval was obtained from the ethical review committee of Arba Minch University, College of Medicine and Health Sciences (IRB/1089/2021/reference). Following the approval, an official letter of co-operation was written to concerned bodies by the Department of Public Health of Arba Minch University. Letter of cooperation was obtained from the respective hospitals and written informed consent was obtained from the study participants after informing the purpose of the study.

## Results

### Socio-demographic characteristics of study participants

Three hundred twelve study participants (156 cases and 156 controls) were included in the study with a 100% response rate. The majority of controls and cases 98(56.4%) and 88(62.8%) were females. The mean age of the respondents was 59±14 (years ±SD) and 57±17(years ±SD) for cases and controls respectively. More than one-third of both cases and controls were in the age group >64 years”. Eighty-nine percent (140) of cases and 137(87.8%) of control were married. Regarding educational background 30(19.2%) of cases and controls had no formal education. Likewise, 89(57.1%) of cases and 95(60.9%) of controls live in an urban area (Table 1).

**Table 1:** Socio-demographic characteristics of diabetic patients under follow-up in clinics at Gamo and Gofa zones, southern, Ethiopia, 2021.

Variables	Case (n(%))	Control (n (%))	Total (n (%))
**Age**			
25–34	6(3.8)	6(3.8)	12(3.8)
35–44	25(16)	16(10.3)	41(13.1)
45–54	38(24.4)	33(21.2)	71(22.8)
55–64	29(18.6)	43(27.6)	72(23.1)
>64	58(37.2)	58(37.2)	116(37.2)
**Marital status**			
Married	140(89.7)	137(87.8)	277(88.8)
Widowed	9(5.8)	8(5.1)	17(5.4)
single	7(4.5)	11(7.1)	18(4.8)
**Sex**			
Male	58(37.2)	68(43.6)	126(40.4)
Female	98(62.8)	88(56.4)	186(50.6)
**Educational status**			
no education	30(19.2)	30(19.2)	60(19.2)
Primary	74(47.4)	68(44.4)	142(45.5)
Secondary	44(28.2)	42(26.1)	86(27.6)
collage and above	8(5.1)	16(10.5)	24(7.7)
**Occupational status**			
Farmer	49(31.4)	52(33.3)	101(32.4)
Housewife	23(11.7)	23(14.7)	46(14.7)
Merchant	33(21.2)	28(17.9)	61(19.5)
Governmental worker	39(25)	36(23.1)	75(24)
Private	12(7.7)	17(10.9)	29(9.3)
**Religion**			
Orthodox	103(66.7)	104(66.7)	207(66.3)
Protestant	43(27.6)	36(23.1)	79(25.3)
Muslim	10(6.4)	16(102)	26(7.7)
**Place of residence**			
Urban	89(57.1)	95 (60.9)	184(59)
rural	67(42.9)	61 (39.1)	128(41)
**Family history of DM**			
Yes	50(32.1)	43 (27.6)	93(29.8)
No	106(67.9)	113(72.4)	219(70.2)
**Income**			
<1500 ETB	40(25.6)	42(26.7)	82(26.3)
1500–3000 ETB	34 (21.8)	38 (24.4)	72(23.1)
>3000 ETB	82(52.6)	76(48.9)	158(50.6)
**Social support**			
Good Supported	104(66.7)	134(85.9)	238(76.3)
Poor supported	52(33.3)	22(14.1)	74(23.7)

Note: ETB-Ethiopian birr; DM-Diabetes Mellitus

### Anti-hyperglycemic medications and poly-pharmacy

Regarding anti-diabetic medications, 77(49.4%) of cases and 73(46.8%) of control were prescribed with Metformin and Glibenclamide followed by metformin alone 28(17.9%) for both cases and controls. Additionally, 35(22.4%) for control and 26(10.7%) for cases take insulin alone. Regarding poly-pharmacy, twenty-one percent of 34(21.8%) of cases and 16(10.3%) of control were taking greater than or equal to five medications including medication for blood glucose control. Regarding the duration of diabetes, the duration of diabetes was greater than 10 years in 32(23.1%) of the cases and 31(19.2%) of the controls. Correspondingly seventy percent of 42(26.9%) of the cases and 60(38.5%) of the controls had abnormal WHR of ≥ 0.9 for males or ≥ 0.85 for females. Regarding comorbidity 77(49.4%) of cases and 46(29.5%) of controls had comorbidity (Table 2).

**Table 2:** Clinical characteristics of adult patients with type 2 diabetes mellitus on follow-up at the diabetic clinic Gamo and Gofa zone public hospitals south Ethiopia, 2021.

Variables	Case (n (%))	Control (n (%))	Total (n (%))
**Type of medication**			
Metformin	28(17.9)	28(17.9)	56(17.9)
Insulin and Metformin	16(10.3)	12(7.7)	28(9)
Metformin and Glibenclamide	77(49.4)	73(46.8)	150(48.1)
Insulin	26(10.7)	35(22.4)	61(19.6)
Glibenclamide	9(5.8)	8(5.1)	17(5.4)
**Poly-pharmacy**			
<5	122(78.2)	140(89.7)	262(84)
>5	34(21.8)	16(10.3)	50(16)
**BMI(kg/m2)**			
Normal	57(36.5)	61(39.1)	118(37.8)
Overweight	44(28.2)	43(27.5)	87(27.8)
Obesity	55(35.3)	52(33.4)	107(34.2)
**WHR**			
Normal	114(73.1)	96(61.5)	210(67.3)
Abnormal	42(26.9)	60(38.5)	102(32.7)
**Duration of diabetes**			
<5yr	59(37.8)	68(43.6)	127(40.7)
5-10yr	61(39.1)	58(37.2)	119(38.1)
>10yr	36(23.1)	30(19.2)	66(21.2)
**Comorbidity**			
Yes	77(49.4)	46(29.5)	123(39.4)
No	79(50.6)	110(70.5)	189(60.6)

Note: BMI-body mass index

### Diabetes self-care activities and behavioral factors

The proportions of T2DM diabetic patients who adhere to dietary recommendations were 47 (30.7%) among cases and 90(57.7%) among controls who had good adherence to the dietary plan. In the assessment of medication adherence, 81(51.9) of cases and 60(38.5%) of controls had no adherence to medication. Of the study participants who did adequate exercise 49(31.4%) percent of the cases and 77(49.4%) of the controls have been involved in, at least 30 min of, exercise for 4 and above- days during the last seven days preceding the study. Moreover, the majority of both cases and controls did not do blood glucose self-monitoring. In addition majority of the study participants never smoked a cigarette and never chew khat in both cases and controls. Regarding alcohol drinking 17(10.9%) of cases and 9(5.8%) of controls were current drinkers (Table 3).

**Table 3:** Diabetes self-care activities of adult patients with type 2 diabetes mellitus on follow-up in a Diabetic clinic of Gamo and Gofa zone public hospitals, southern, Ethiopia, 2021.

Variables	Case (n (%))	Control (n (%))	Total no (n (%))
**DM association membership**			
Yes	45(28.8)	58(32.7)	103(33)
No	111(71.2)	98 (62.8)	209(67)
**Blood glucose self-monitoring**			
Yes	27(17.3)	42(26.9)	69(22.1)
No	129 (82.7)	114(73.1)	243(77.9)
**Cigarette smoking**			
Never	144(92.3)	140(89.7)	284(91)
Past	6(3.8)	8(5.1)	14(4.5)
Current	6(3.8)	8(5.1)	14(4.5)
**Alcohol drinking**			
Never	104 (66.7)	104(66.7)	208(66.7)
Past	35(22.4)	43(27.6)	78(25)
Current	17(10.9)	9(5.8)	26(8.3)
**Khat chewing**			
Never	134(85.9)	131(84)	265(84.7)
Past	12(7.7)	14(9)	26(8.3)
Current	10(6.4)	11(7.1)	21(6.7)
**Physical exercise**			
Adequate	49(31.4)	77(49.4)	126(40.4)
Not adequate	107(68.6)	79(50.6)	186(69.6)
**Dietary adherence**			
Adhere	47(30.1)	90(57.7)	137(43.9)
Not adhere	109(69.9)	66(42.3)	175(56.1)
**Medication adherence**			
Adhere	75(48.1)	96(61.5)	171(54.8)
Not adhere	81(51.9)	60(38.5)	141(45.2)

### Factors associated with poor glycemic control

In bi-variable logistic regression analysis, educational level, blood glucose self-monitoring, being a member of DM association, social support, comorbidity, dietary adherence, medication adherence, poly-pharmacy, sex, physical exercise, and alcohol drinking were found to have p-value <0.25 and entered into the multivariate logistic analysis. In multivariable binary logistic analysis, poly-pharmacy, dietary adherence, comorbidity, physical exercise, and social support were found to be significantly associated with poor blood sugar control (Table 4).

**Table 4:** Bivariate and multivariate logistic regression of factors associated with poor glycemic control among type 2 diabetic patients under follow-up clinics at Gamo and Gofa zones, Southern, Ethiopia, 2021.

Variables	Cases (n (%))	Controls (n (%))	COR (95% CI)	AOR (95% CI)
**Comorbidity**				1.145
				3.425
				1.981
				1.145
				3.425
Yes	77(49.4)	46(29.5)	2.33(1.46–3.71)*	2.35(1.39–3.95)** 1.399
				**3.949**
				2.06(1.20–3.54)**
No	79(50.6)	110(70.5)	**1**	**1**
**Poly-pharmacy**				
<5	122(78.2)	140(89.7)	**1**	**1**
>5	34(21.8)	16(10.3)	2.44(1.28–4.63)*	2.83(1.39–5.74)**
**Physical Exercise**				
Adequate	49(31.4)	77(49.4)	1	11
Inadequate	107(68.6)	79(50.6)	2.13(1.34–3.38)*	1.86(1.11–3.12)**
**Social support**				
Good support	104(66.7)	134(85.9)	**1**	**1**
Poor support	52(33.3)	22(14.1)	3.04(1.74–5.33)*	3.05 (1.64–5.65)**
			**5.334**	
**Self-blood glucose monitoring**				
Yes	27(17.3)	42(26.9)	1	1
No	129 (82.7)	114(73.1)	.57(.33-.98)*	1.48(.77–2.84)
**Medication Adherence**				
Adhere	75(48.1)	96(61.5)	1	1
Un-adhere	81(51.9)	60(38.5)	1.73(1.10–2.77)*	1.16(.66–2.04)
**Being a member of the DM association**				
Yes	45(28.8)	58(32.7)	1	1
No	111(71.2)	98 (62.8)	1.46(.91–2.35)*	.67(.38–1.17)
**Educational status**				
No education	30(19.2)	30(19.2)	1	1
Primary	74(47.4)	68(44.4)	1.09(.59–1.99)	1.17(.55–2.48)
Secondary	44(28.2)	42(26.1)	1.05(.54–2.03)	.84(.38–1.88)
Collage and above	8(5.1)	16(10.5)	.50(.19–1.34)*	.69(.21–2.20)
**Sex**				
Male	58(37.2)	68(43.6)	1	1
Female	98(62.8)	88(56.4)	1.30(.83–2.05)*	.67(.38–1.16)
**Dietary adherence**				
Adhere	47(30.7)	90(57.7)	0.32(0.19–0.50)*	.31(.19-.51)***
Not adhere	109(69.3)	66(42.3)	1	1
**Alcohol drinking**				
Never	104 (66.7)	104(66.7)	1	1
Past	35(22.4)	43(27.6)	.81(.48–1. 37)*	.83(.44–1.56)
Current	17(10.9)	9(5.8)	1.88(.80–4.43)*	1.10(.37–3.32)

Note: * Significant at P-value≤0.25; **Significant at P-Value <0.05; COR–Crude Odds Ratio; AOR–Adjusted Odds Ratio

The odds of poor glycemic control were 2.35 times higher among T2DM with comorbidity compared to those who do not have comorbidity (AOR = 2.35, 95% CI (1.39–3.95)). The odds of poor glycemic control were lower for those T2DM diabetic patients who adhere to a dietary recommendation by 69% compared to those T2DM diabetic patients who do not adhere to a dietary recommendation (AOR = 0.31, 95% CI (.19-.51)) (Table 4).

The odds of poor glycemic control were 2.83 times higher among T2DM with poly-pharmacy as compared to participants who had no ploy pharmacy (AOR = 2.83, 95% CI (1.37–5.85)). The odds of poor glycemic control were1.86 times (AOR = 1.86 95% CI (1.11–3.12)) higher among T2DM patients involved in physical activity only for less than three days as compared to participants doing regular physical activity for more than three days.

The odds of poor glycemic control were 2.35 times higher among T2DM with comorbidity compared to those who do not have comorbidity (AOR = 2.35, 95% CI (1.39–3.95)). The odds of poor glycemic control were lower for those T2DM diabetic patients who adhere to a dietary recommendation by 69% compared to those T2DM diabetic patients who do not adhere to a dietary recommendation (AOR = 0.31, 95% CI (.19-.51)) (Table 4).

## Discussion

This study assessed factors associated with poor glycemic control among T2DM patients in Gamo and Gofa zone public hospitals. The results show that comorbidity, poly-pharmacy, social support, dietary adherence, and physical exercise are factors associated with poor glycemic control.

In this study, the odds of poor glycemic control were 3.05 times higher among T2DM patients with poor social support compared to those with good social support (AOR = 3.05, 95% CI (1.64–5.65)). In line with this study finding a study in China, Ghana, New work also revealed that Social support had significant correlations with glycemic [35–37]. The possible justification can be diabetic patients who have low social support have low self-care practice which indirectly leads to poor glycemic control [38]. Increased social support is directly associated with reduced HbA1c, but it is also indirectly associated with HbA1c through various mechanisms including diet and medication adherence. Social support is important in helping patients with diabetes cope with the disease and improves treatment adherence. In addition results in turkey indicate that social support and empowerment are important for nurses to consider when planning interventions that increase the self-care behavior of individuals with type 2 diabetes and for improving glycemic control [26]. A study was done in turkey also revealed that Social support was a statistically significant predictor of, blood glucose monitoring (42). In contrast, a study done in South Africa revealed that there was no association between social support and HbA1c this might be because the population might be different in way of life and culture [39].

In this study, the odds of poor glycemic control were 2.83 times higher among T2DM patients with poly-pharmacy as compared to participants who had no ploy pharmacy (AOR = 2.83, 95% CI (1.39–5.74)). A possible justification can be Poly-pharmacy increases the probability of adverse drug events [40, 41]. Therefore poly-pharmacy might decrease compliance to anti-diabetic medications and leads to suboptimal glycemic control [42, 43]. A study done in Romania revealed that T2DM patients who received more drugs than their non-diabetes counterparts were exposed to more drug-drug and food-drug interactions [43]. And another possible justification can be low medication adherence to prescribed medications.

The odds of poor glycemic control were 1.86 times higher among T2DM patients with involvement in physical activity only for less than three days as compared to participants doing regular physical activity for more than three days (AOR = 1.86 95% CI (1.11–3.12)). This is in line with the study done in Jimma, Nekemte, Saudi Arabia, Jordan, and Thailand [15, 44–47]. This might be because, in people with type 2 diabetes, exercise can improve peripheral insulin sensitivity as well as enhance insulin binding. Exercise also decreases abdominal fat, reduces free fatty acids, and increases insulin-sensitive skeletal muscle, which may result in improved glycemic control [48, 49].

In this study, the odds of poor glycemic control were 2.35 times higher among T2DM patients with comorbidity when compared to those T2DM patients who do not have comorbidity (AOR = 2.35, 95% CI (1.39–3.95)). This finding is consistent with studies done in Mekelle town, Jimma, India, and the Netherlands [15, 50–52]. The possible justification might be because the presence of comorbid illness aggravates disease processes and reduces their quality of life [53]. Another reason might be comorbidity increases poly-pharmacy which increases pill burden and adverse drug reactions. In contrast with this, a study at a police referral hospital in Addis Ababa Ethiopia 2018 revealed that the presence of comorbidity showed a high likelihood of FPG target achievement among the patients this might be because the study participants might have good adherence to medication than comorbid T2DM diabetic study participants in this study [54].

Finally, In this study, the odds of poor glycemic control were lower for those T2DM patients who adhere to a dietary recommendation by 69% compared to those type 2 diabetic patients who do not adhere to dietary recommendations (AOR = .31, 95% CI (.19-.51)). This finding is consistent with the studies conducted at Suhul Hospital, Haromaya, Tigray, Debre-Tabour, Ethiopia, which determined that good dietary adherence is significantly associated with having good glycemic control [16, 23, 54, 55]. A possible justification could be avoiding a high-fat diet, especially one high in saturated fats, which has been linked to T2DM and insulin resistance. It appears that saturated fatty acids (but not unsaturated fats) activate immune cells, which produce an inflammatory protein, which in turn then makes cells more insulin resistant so then avoiding a high-fat diet decreases the occurrence of insulin resistance. And also Adherence to diet facilitate weight loss, improving glucose control and lipid profiles in patients with T2DM [56].

## Conclusion and recommendation

In this study, the presence of comorbidity, physical exercise, poly-pharmacy, low social support, and adherence to dietary recommendations were factors associated with poor glycemic control. Therefore, concerned health authorities and health professionals should give special attention to regular follow-up and control of blood glucose levels. Healthcare providers should take aware of psychosocial factors in the treatment regime of the patient. Family members and society should be educated about diabetes, the importance of controlling blood glucose levels consciously, and the long-term complications of the disease. A cohort study is recommended for future researchers to infer substantial evidence of causality.

## Limitations of the study

None of the patients had HbA1c determination due to the unavailability of the laboratory service for the HbA1c determination in the study hospitals. As some parts of the questionnaire depended on the memory of respondents may have resulted in recall bias.

## List of abbreviations

ADA: American Diabetes Association
AOR: Adjusted Odds Ratio
CI: Confidence Interval
DM: Diabetes Mellitus
DKA: Diabetic Ketoacidosis
DGT: Decreased Glucose
ETB: Ethiopian Birr
FPG: Fasting Plasma Glucose
FGC: Fasting Glucose Change Tolerance
GTT: Glucose Tolerance Test
GD: Gestational Diabetes
HC: Hip Circumference
IDF: International Diabetes Federation
MMS: Modified Morisky Scale
T1DM: Type 1 Diabetes Mellitus, T2D: Type 2 Diabetes
T2DM: Type 2 Diabetes Mellitus, SDCA: Self Diabetic Care Activity
SPSS: Statistical Package for the Social Sciences
NCD: Non-Communicable Diseases
OPD: Outpatient Department
WC: Waist Circumference
WHO: World Health Organization
